# LHX2 promotes malignancy and inhibits autophagy via mTOR in osteosarcoma and is negatively regulated by miR-129-5p

**DOI:** 10.18632/aging.102427

**Published:** 2019-11-13

**Authors:** Honghai Song, Jiaming Liu, Xin Wu, Yang Zhou, Xuanyin Chen, Jiangwei Chen, Keyu Deng, Chunxia Mao, Shanhu Huang, Zhili Liu

**Affiliations:** 1 Department of Science and Technology, The First Affiliated Hospital of Nanchang University, Nanchang, Jiangxi 330006, China; 2 Department of Orthopedic Surgery, The First Affiliated Hospital of Nanchang University, Nanchang, Jiangxi 330006, China; 3 Jiangxi Institute of Respiratory Disease, The First Affiliated Hospital of Nanchang University, Nanchang, Jiangxi 330006, China; 4 The National Engineering Research Center for Bioengineering Drugs and Technologies, Institute of Translational Medicine, Nanchang University, Nanchang, Jiangxi 330031, China

**Keywords:** osteosarcoma, LHX2, autophagy, mTOR pathway, miR-129-5p

## Abstract

The transcript factor LHX2 is dysregulated in many cancers but its role in osteosarcoma (OS) remains unclear. In this study, we confirm that LHX2 is up-regulated in osteosarcoma, and that its silencing inhibits OS malignancy and induces autophagy via mTOR signaling. We further demonstrate that miR-129-5p negatively regulates LHX2 and suppresses the malignant phenotypes of OS. LHX2 overexpression could restore the malignant phenotypes. In conclusion, LHX2 regulates tumorigenesis and autophagy via mTOR in OS and is negatively regulated by miR-129-5p. Targeting the miR-129-5p/LHX2/mTOR axis therefore represents a novel therapeutic strategy for OS treatment.

## INTRODUCTION

Osteosarcoma (OS) is a primary solid tumor of the bone that occurs in children and the elderly [1, 2]. Multi-agent chemotherapy regimens have improved 5-year event-free survival (EFS) rates from less than 20%, to 65% ~70% in patients with no metastasis [3]. However, for patients with metastasis at initial diagnosis or with recurrent disease, the 5-year EFS is lower than 20% [3, 4]. In the last 30 years, considerable progress has been made to understand the pathogenesis of human OS. New therapies for both localized and metastatic disease are urgently required.

LHX2, also known as LH2, is a member of the LIM family consisting of two zinc finger domains. LHX2 participates in cell differentiation, embryonic development and hair formation [5–7]. Emerging evidence demonstrates that LHX2 is an oncogene in various tumors. LHX2 promotes the growth and metastasis of nasopharyngeal carcinoma by regulating Wnt signaling [8]; facilitates breast cancer metastasis through PDGF-B signaling [9]; and is aberrantly expressed in lung cancer and pancreatic ductal carcinoma [10, 11]. Despite this knowledge, the role of LHX2 in osteosarcoma remains elusive.

Macroautophagy/autophagy is a highly conserved cellular process. The primary function of autophagy is to degrade superfluous or damaged organelles through lysosomes [12]. Autophagy plays a dual role in cancer [13]. On the one hand, cells support cell metabolism, avoid death and promote metastasis by activating self-protective autophagy to maintain cell viability under starvation or suspension [14, 15]. On the other hand, autophagic cell death, also defined as type-2 programmed cell death, is typically related to tumor growth, metastasis and the drug resistance of cancer cells [16–18].

mTOR signaling controls cell proliferation and migration [19, 20]. The aberrant activation of mTOR enhances the tumorigenesis of cancer cells. In addition, mTOR signaling is also a critical regulator of autophagy [21, 22], and akt-mTOR activation prevents the phosphorylation of ULK1 (Ser757) under nutrient sufficiency [23]. This coordinated phosphorylation is essential for ULK1-mediated autophagy induction.

MiRNAs are 18 to 25 nucleotide, small non-coding RNAs that participate in a variety of cellular processes through their binding to the 3'UTR of target mRNAs and leading to translational inhibition of the target genes [24]. Studies have reported that miRNAs play a crucial role in tumorigenesis [25, 26], including miRNA-129-5p, the abnormal expression of which has been detected in breast cancer [27], lung cancer [28] and glioma [29]. Our previous studies demonstrated that miR-129-5p suppresses the malignant phenotypes of OS [30]. In this study, we demonstrate that LHX2 is an oncogene during OS progression through its regulation of the mTOR pathway. We further highlight LHX2 as a novel target of miR-129-5p.

## RESULTS

### LHX2 is up-regulated in osteosarcoma and predicts poor prognosis

**Figure 1:**
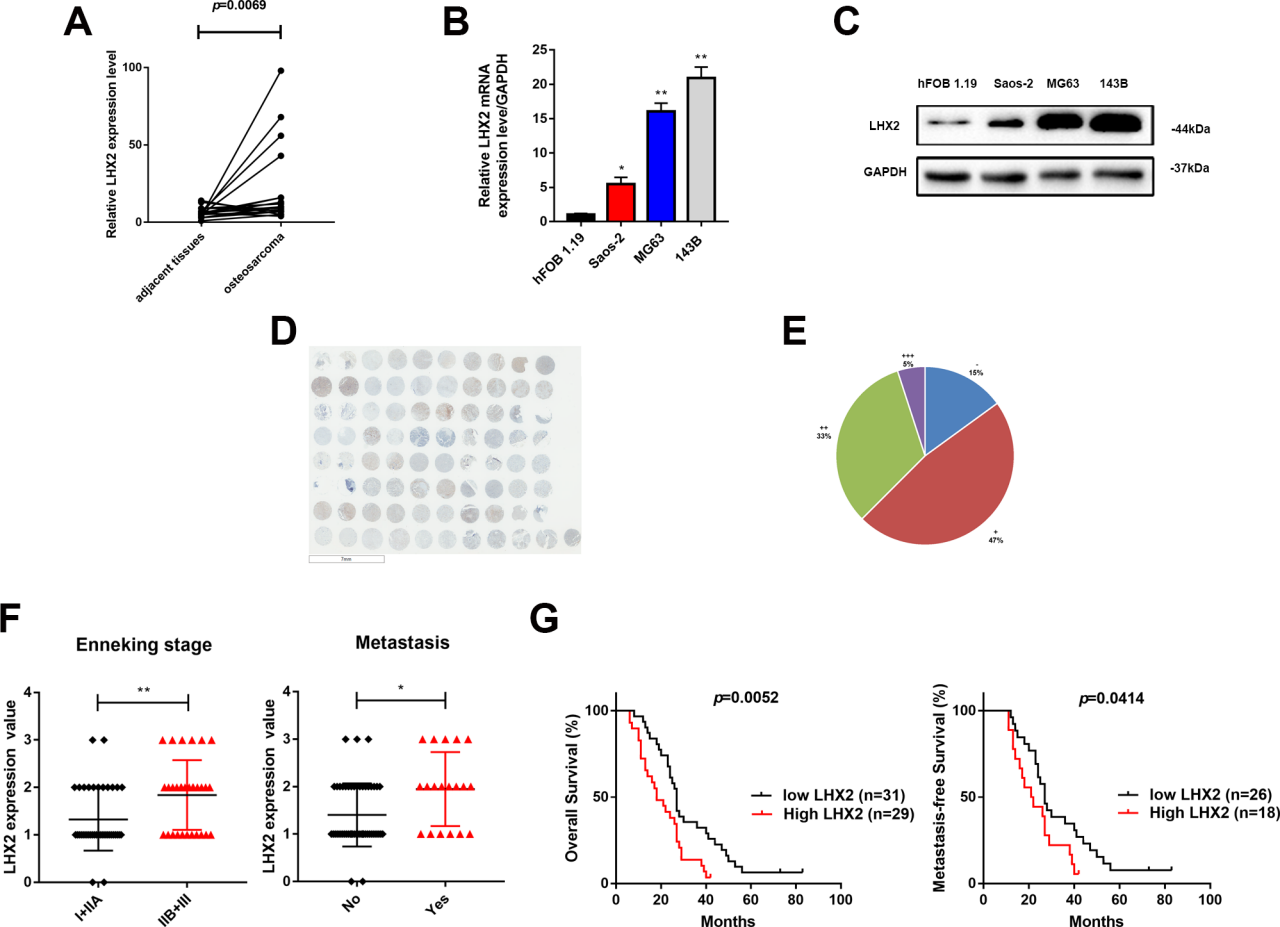
**LHX2 is up-regulated in osteosarcoma and predicts poor prognosis.** (**A**) Comparison of LHX2 expression in 18 paired OS and non-tumor tissues in the GEO database. **P <0.01. (**B**) LHX2 mRNA levels in OS cell lines. *P <0.05, **P<0.01. (**C**) LHX2 protein levels in OS cell lines. LHX2 expression is relative to GAPDH. (**D**) IHC staining of LHX2 in OS TMA (n=40, double dots per case). (**E**) Statistical analysis of IHC staining in human OS TMA (n=40). (**F**) Distribution of LHX2 IHC staining scores in OS tissues according to distant metastasis and Enneking stage classification. (**G**) Kaplan-Meier survival analysis according to LHX2 expression.

Previous studies have demonstrated that LHX2 functions as an oncogene during tumor development [9–11], but the expression and function of LHX2 in OS remain unknown. Bioinformatics were thus performed to explore the expression of LHX2 in OS samples. From 18 primary OS and paired adjacent normal tissues in the GEO database, LHX2 expression was significantly higher in OS tissues (*p*=0.0069) (Figure 1A). High LHX2 expression levels in OS cell lines were also detected. As shown in Figure 1B and 1C, both the mRNA and protein levels of LHX2 were significantly higher in Saos-2, 143B and MG63 cell lines compared to hFoB 1.19 cells. We further detected LHX2 levels in OS tissue microarrays (TMA) through immunohistochemistry (IHC) (Figure 1D). LHX2 was detected in the majority of samples (34/40, 85%), 47% (19/40) of which had an IHC score of (+), 33% (13/40) had an IHC score of (++), and 5% (2/40) had an IHC score of (+++) (Figure 1E). We next assessed the relationship between clinical parameters and LHX2 expression in 71 OS patients. The results revealed that LHX2 closely correlates with pulmonary metastasis and Enneking stage (Figure 1F; Table 1). Moreover, Kaplan-Meier (KM) analysis revealed that high LHX2 levels were associated with poor overall survival (*P*=0.0052) and metastasis-free survival (*P*=0.0414) (Figure 1G) in OS patients. These results indicate that LHX2 as an oncogene in OS.

**Table 1:** Correlation of LHX2 protein expression levels in OS tissues with clinical pathologic parameters.

**Variables**	**All cases**	**LHX2 expression**		**P-value**
		**Low**	**High**	
Gender				
Male	41	20 (48.8%)	21 (51.2%)	0.349
Female	30	18 (60%)	12 (40%)	
Age				
≤20	35	20 (57.1%)	15 (42.9%)	0.358
>20	36	18 (50%)	18 (50%)	
Location				
Femur/Tibia	54	27 (50%)	27 (50%)	0.289
Elsewhere	17	11 (64.7%)	6 (35.3%)	
Tumor size (cm)				
≤5	23	15 (65.2%)	8 (34.8%)	0.133
>5	48	23 (47.9%)	25 (52.1%)	
Distant metastasis				
Yes	52	29 (55.8%)	23 (44.2%)	0.013^*^
No	19	6 (31.6%)	13 (68.4%)	
Enneking staging				
I+IIA	40	28 (70%)	12 (30%)	0.007^**^
IIB+III	31	10 (32.3%)	21 (67.7%)	

*P*-value was calculated by Pearson’s Chi-Square test. *P <0.05, **P<0.01.

### LHX2 silencing decreases the proliferation, migration and invasion of OS cells

**Figure 2:**
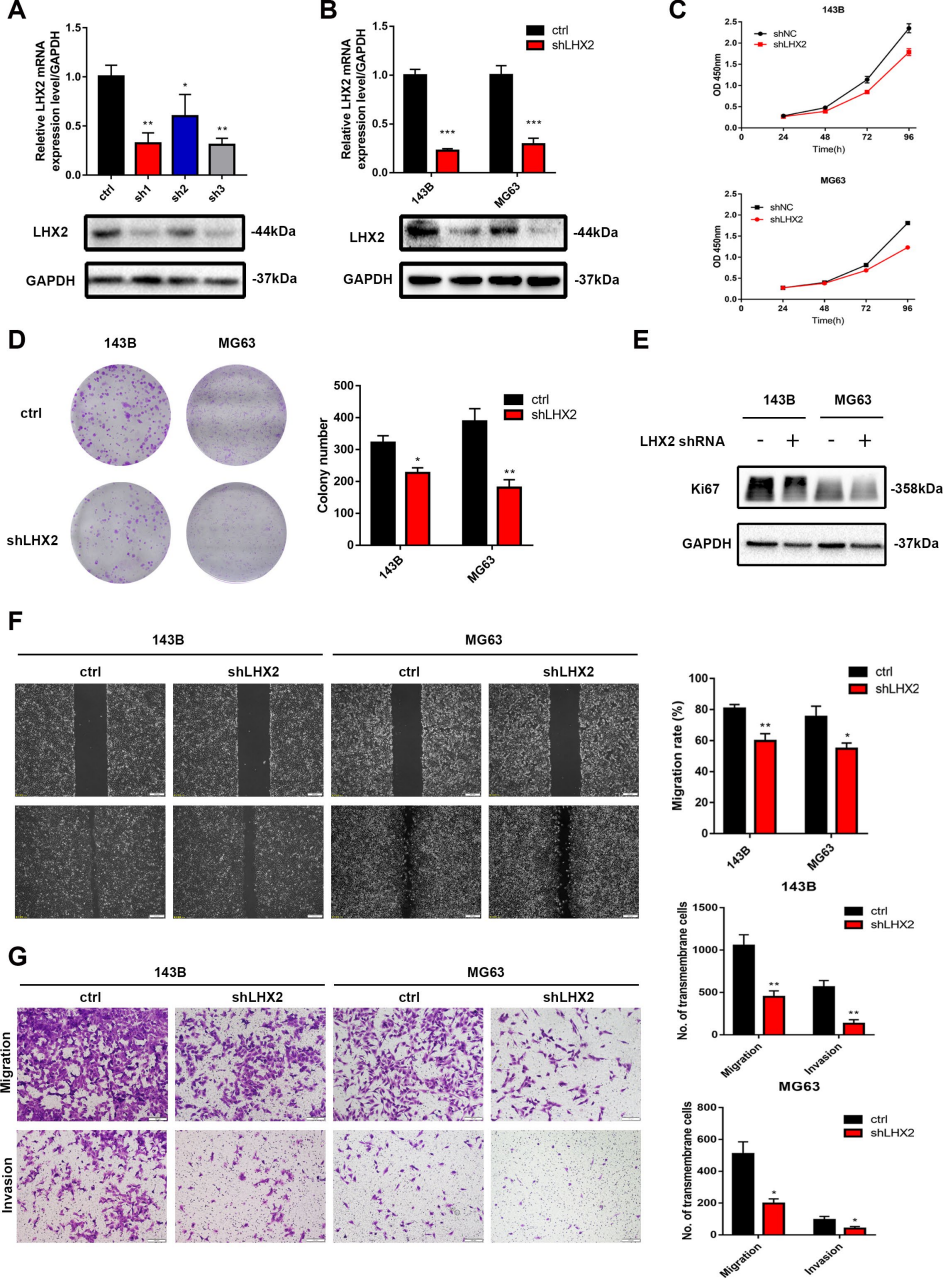
**LHX2 silencing decreases the proliferation, migration and invasion of OS cells.** (**A**) qRT-PCR and WB analysis for the knockdown efficiency of LHX2 using three different short hairpins. (**B**) qRT-PCR and WB analysis for LHX2 silencing efficiency. (**C**–**D**) CCK8 and colony formation assays for 143B and MG63 cells after LHX2 silencing. (**E**) Western blot analysis of Ki67 expression. (**F**) Representative images of wound healing assays; cell migration rates were measured after 24 h. Scale bar: 100 μm. (**G**) Representative images of transmembrane cells in shLHX2 and control groups. *P <0.05, **P<0.01. Scale bars: 200 μm.

To explore the role of LHX2 inhibition on OS malignant phenotypes, endogenous LHX2 levels were silenced using three shRNAs (short-hairpin RNA) (Figure 2A–2B) and the effect on OS cell proliferation was assessed via CCK8 assays, colony formation assays and Ki67 expression. We found that LHX2 silencing significantly decreased proliferation, colony numbers (Figure 2C–2D) and Ki67 expression (Figure 2E). These findings indicated that LHX2 silencing suppresses OS cell proliferation. To confirm these findings, wound healing and transwell assays were performed to investigate the role of LHX2 inhibition in the migratory and invasive ability of OS cells. The results indicated that LHX2 silencing significantly reduced the migration rates and invasive ability of all OS cell lines assessed (Figure 2F–2G). To exclude the impact of the off-target effect, we transfected another two effective shRNAs (shLHX2-1 and shLHX2-2) into 143B and MG63 cells, and representative assays were repeated to assess the biological function of LHX2. The results were consistent with what we mentioned above (Supplementary Figure 1A–1C).

### LHX2 knockdown enhances autophagy and inhibits mTOR signaling pathway in vitro

**Figure 3:**
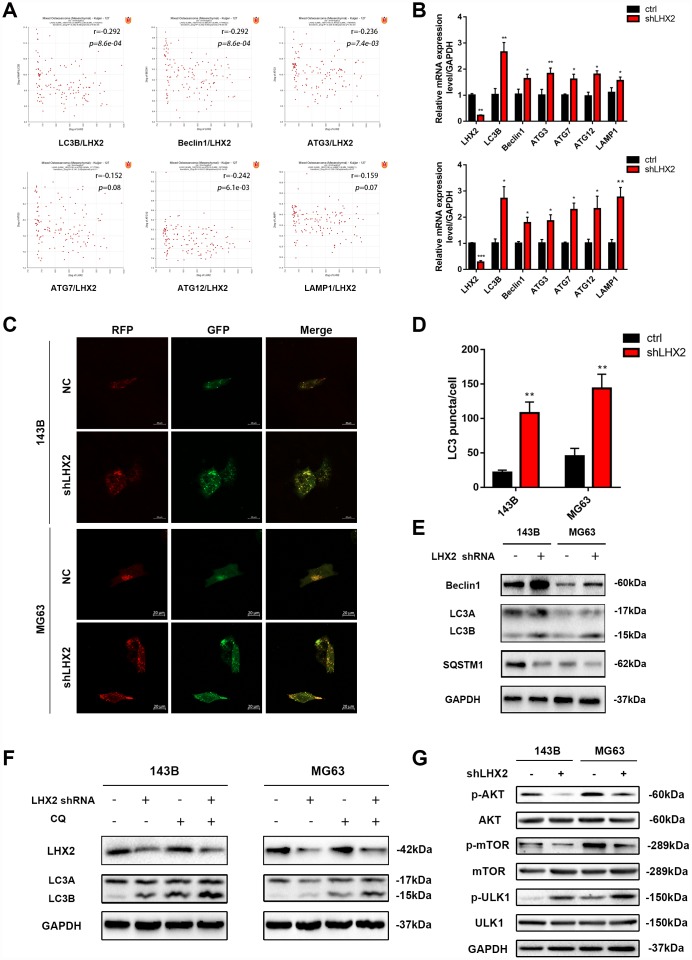
**LHX2 knockdown enhances autophagy and inhibits mTOR signaling *in vitro*.** (**A**) Pearson correlation analysis between LHX2 and autophagy-related genes in 127 OS patients. (**B**) qRT-PCR analysis of autophagy-related gene expression in LHX2 knockdown cells. *P <0.05, **P<0.01. (**C**) Representative confocal images of LC3 in lv-GFP-RFP-LC3–infected 143B and MG63 cells. Scale bar: 20 μm (**D**) LC3 puncta in each cell were counted under 100× magnification. **P<0.01. (**E**) Autophagy-related protein (LC3B, Beclin1, SQSTM1) levels in lv-control or lv-shLHX2-infected 143B and MG63 cells. (**F**) 143B and MG63 cells were treated with or without CQ (20 μM) for 12 h. LHX2 and LC3B proteins were detected by western blot. (**G**) Western blots of p-Akt (Ser473), Akt, p-mTOR (Ser2448), mTOR, p-ULK1 (Ser757), ULK1 in 143B and MG63 cells.

Whilst LHX2 inhibition was shown to suppress the malignant phenotypes of OS cells, its effects on autophagy remained unclear. To investigate this, the bioinformatic prediction tool R2 was used to investigate the relationship between LHX2 and autophagic proteins in OS tissues. The results showed that LHX2 expression was negatively associated with known biomarkers of autophagy (Beclin1, LC3B, ATG3, ATG7, ATG12, and LAMP1) (Figure 3A). qRT-PCR analysis also confirmed that LHX2 silencing could increase the mRNA levels of the autophagy-related genes Beclin1, LC3B, ATG3, ATG7, ATG12, and LAMP1 in the OS cell lines 143B and MG63 (Figure 3B). To monitor the formation of autophagosomes and autolysosomes in LHX2-silenced cells, cells were infected with lv/RFP-GFP-LC3. As shown in Figure 3C–3D, the number of red LC3 puncta (representing autolysosomes) and yellow LC3 puncta (representing autophagosomes) were enhanced in 143B and MG63 cells, indicated increased autophagic flux. Western blot analysis revealed that the ratio of LC3B to LC3A and the levels of Beclin1 increased, whilst the autophagy marker p62 (SQSTM1) also decreased when LHX2 was silenced (Figure 3E). The accumulation of lipidated LC3 and autophagosomes results from either autophagy induction or the exhaustion of autophagic flux. To discriminate these distinct mechanisms and evaluate the role of LHX2 on autophagic processes, cells were treated with the specific autophagy inhibitor chloroquine (CQ), which prevents LC3 degradation by inhibiting the fusion of autophagosomes with lysosomes. As shown in Figure 3F, the levels of LC3B remarkably increased following CQ treatment in both control and LHX2-silenced 143B and MG63 cells compared to untreated groups. This suggested that LHX2 silencing induces autophagic flux, but not inhibits autophagy.

Akt/mTOR signaling regulates tumor progression and autophagy [22, 23, 31]. To explore the molecular mechanisms of LHX2 during tumor progression, we assessed several critical regulators of the Akt/mTOR pathway. Western blot analysis revealed that LHX2 silencing did not alter total Akt, mTOR and ULK1 levels, but phosphorylated p-Akt (Ser473) and p-mTOR (Ser2448) levels significantly declined. In addition, the levels of p-ULK1 (Ser757) were increased in LHX2 silenced 143B and MG63 cells (Figure 3G). Similar results were obtained in 143B and MG63 cells transfected with another two shRNAs (Supplementary Figure 1D and 1E). This suggests that LHX2 silencing inhibits malignant phenotypes and promotes autophagy through mTOR.

### LHX2 silencing inhibits OS growth and metastasis in vivo

**Figure 4:**
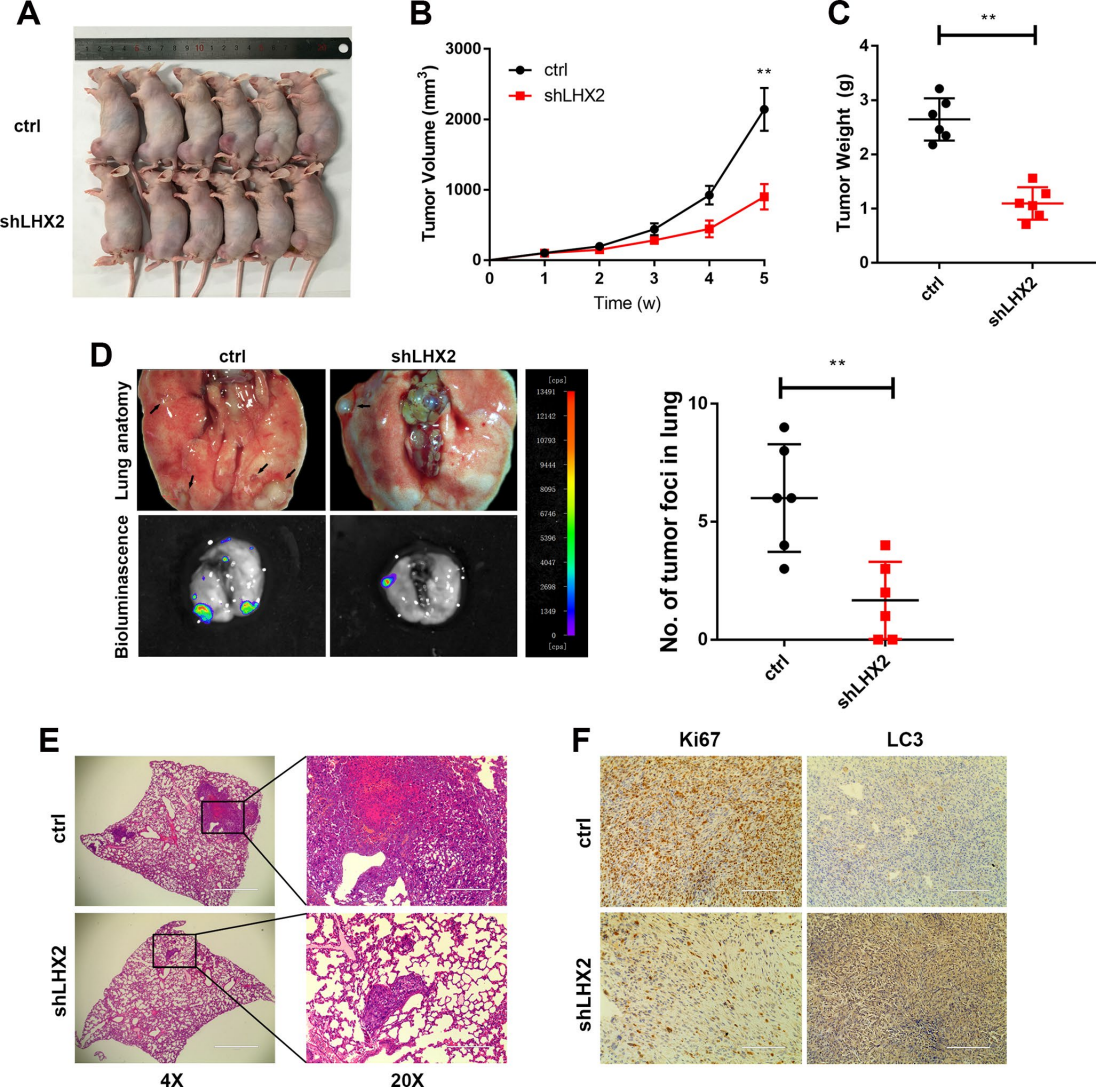
**LHX2 silencing inhibits OS growth and metastasis in vivo.** (**A**) OS-143B cells stably expressing luciferase and the indicated genes were inoculated into nude mice (n=6), and mice were sacrificed after 5 weeks. (**B**) Tumor sizes were measured weekly and calculated using the following formula: *V*= (Length×Width^2^/2). (**C**) Orthotopic tumors were dissected and weighted. **P<0.01. (**D**) Representative bioluminescence and lung anatomy of the metastasis of OS cells, black arrows indicate possible metastatic lesions. Metastatic foci in the lungs were determined by luminescence signals and counted. **P<0.01. (**E**) Representative H&E staining of lung sections. (**F**) IHC analysis of Ki67 and LC3 in tumors from tumor-bearing mice. Scale bar: 100 μm.

To determine the effects of LHX2 *in vivo*, orthotopic xenograft models were established using 143B cells tagged with luciferase. Mouse tumors treated with Lv-shLHX2 showed significantly suppressed tumor growth (Figure 4A–4C). Bioluminescent imaging system was used to detect pulmonary metastases. The number of tumor foci in the lungs significantly decreased when LHX2 expression was silenced. Similar results were observed in lung H&E stained sections (Figure 4D–4E). Furthermore, IHC analysis showed higher LC3B expression in OS tissue, whilst the IHC score of the cell proliferation marker Ki67 was lower in the shLHX2 group (Figure 4F). These results suggested that the inhibition of LHX2 could inhibit OS tumor growth and lung metastasis *in vivo*.

### LHX2 is directly targeted by miR-129-5p in OS cells

**Figure 5:**
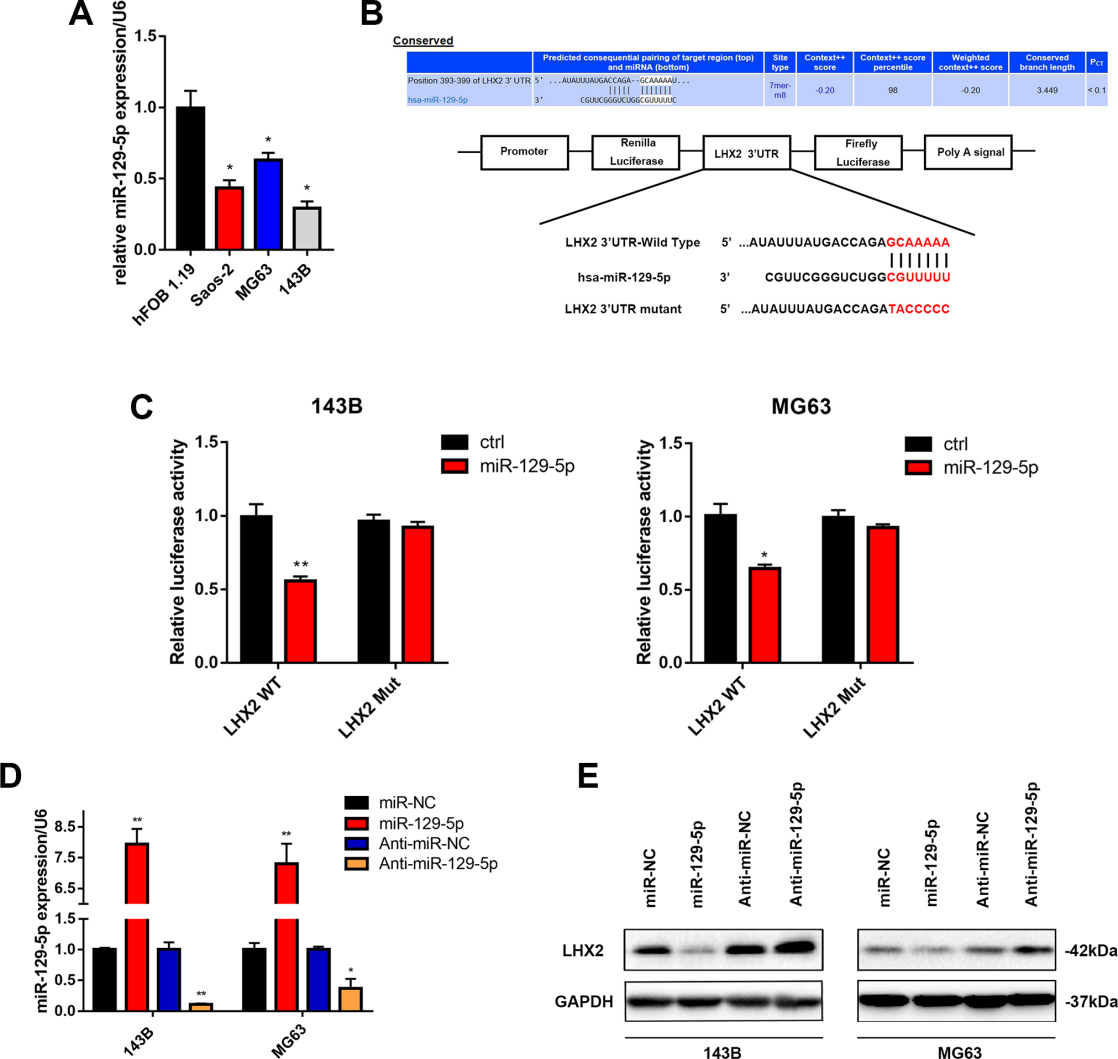
**LHX2 is directly targeted by miR-129-5p in OS cells.** (**A**) miR-129-5p expression in OS cell lines determined by qPCR. *P <0.05. (**B**) miR-129-5p binding was predicted in the 3’UTR of LHX2 using the informatic tool Targetscan. (**C**) Dual-luciferase reporter assays were performed in 143B and MG63 cells cotransfected with putative or mutant LHX2 3’UTR-luciferase reporters and lv-miR-129-5p. *P <0.05, **P<0.01. (**D**) Corresponding expression of miR-129-5p for 143B and MG63 cells infected with lv-miR-129-5p or lv-Anti-miR-129-5p. *P <0.05, **P<0.01. (**E**) LHX2 expression in OS cells infected with lv-miR-129-5p or lv-Anti-miR-129-5p.

To explore the potential mechanisms influencing the stability of LHX2 mRNA, we screened two bioinformatic tools Targetscan and miRanda to select a potential upstream of LHX2. In our previous studies, we found that miR-129-5p participates in OS development [30]. We hypothesized that miR-129-5p inhibits malignancy through the suppression of LHX2 production in OS cell lines. To verify this, we examined miR-129-5p expression levels in OS cells and observed its down-regulation in OS cells (Figure 5A). Next, we transiently co-transfected luciferase reporter constructs with mutated or putative LHX2 3′-UTRs into 143B and MG63 cell lines to evaluate whether LHX2 is directly targeted by miR-129-5p (Figure 5B). miR-129-5p overexpression inhibited LHX2 3′-UTR reporter activity but had no significant effects on luciferase activity when the binding sites were mutated in 143B and MG63 cells (Figure 5C). Consistent with these results, western blot analysis indicated that miR-129-5p decreased the expression of LHX2. In contrast, the inhibition of miR-129-5p promoted the expression of LHX2 in both 143B and MG63 cells (Figure 5D and 5E). These results indicate that miR-129-5p downregulates LHX2 by directly targeting the LHX2 3′-UTR *in vitro*.

### LHX2 restoration partially reverses miR-129-5p overexpression-mediated malignant phenotypes in vitro

**Figure 6:**
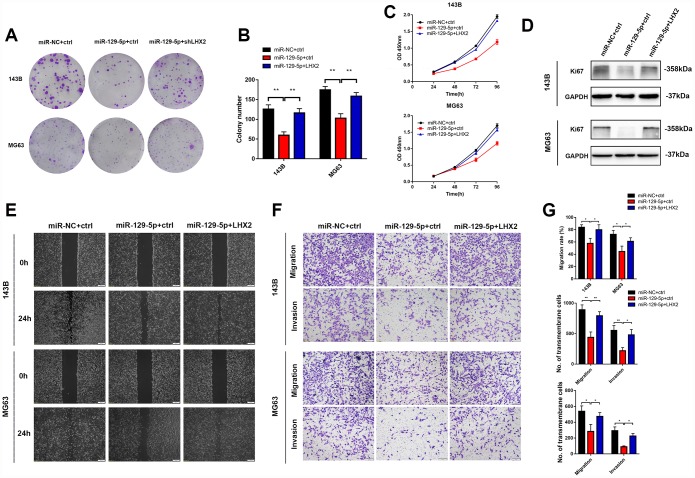
**LHX2 restoration partially reverses miR-129-5p overexpression-mediated malignant phenotypes in vitro.** (**A**–**B**) CCK8 and colony formation assays indicated that the inhibitory effects of miR-129-5p can be partially reversed by LHX2 overexpression. **P<0.01. © Ki67 expression in shLHX2 and control groups were determined by western blot. (**D**) Represent imaging of wound healing assays and cell migration rates were measured after 24 h. Scale bar: 100 μm. (**E**) Represent images of transmembrane cells in shLHX2 and control groups. *P <0.05, **P<0.01. Scale bars: 200 μm. (**F**) Cell migration rates and the number of transmembrane cells.

To evaluate the effects of miR-129-5p on the malignant phenotypes of OS, 143B and MG63 cells were infected with lv-miR-129-5p and proliferation, migration and invasion were assessed. We found that miR-129-5p suppresses the proliferation and migration of both cell lines which could be partially rescued by LHX2 overexpression (Figure 6A–6I). Collectively, these data suggest that miR-129-5p inhibits OS malignant phenotypes through the suppression of LHX2.

### Restoration of LHX2 reverses miR-129-5p overexpression-mediated malignant phenotypes in vivo

**Figure 7:**
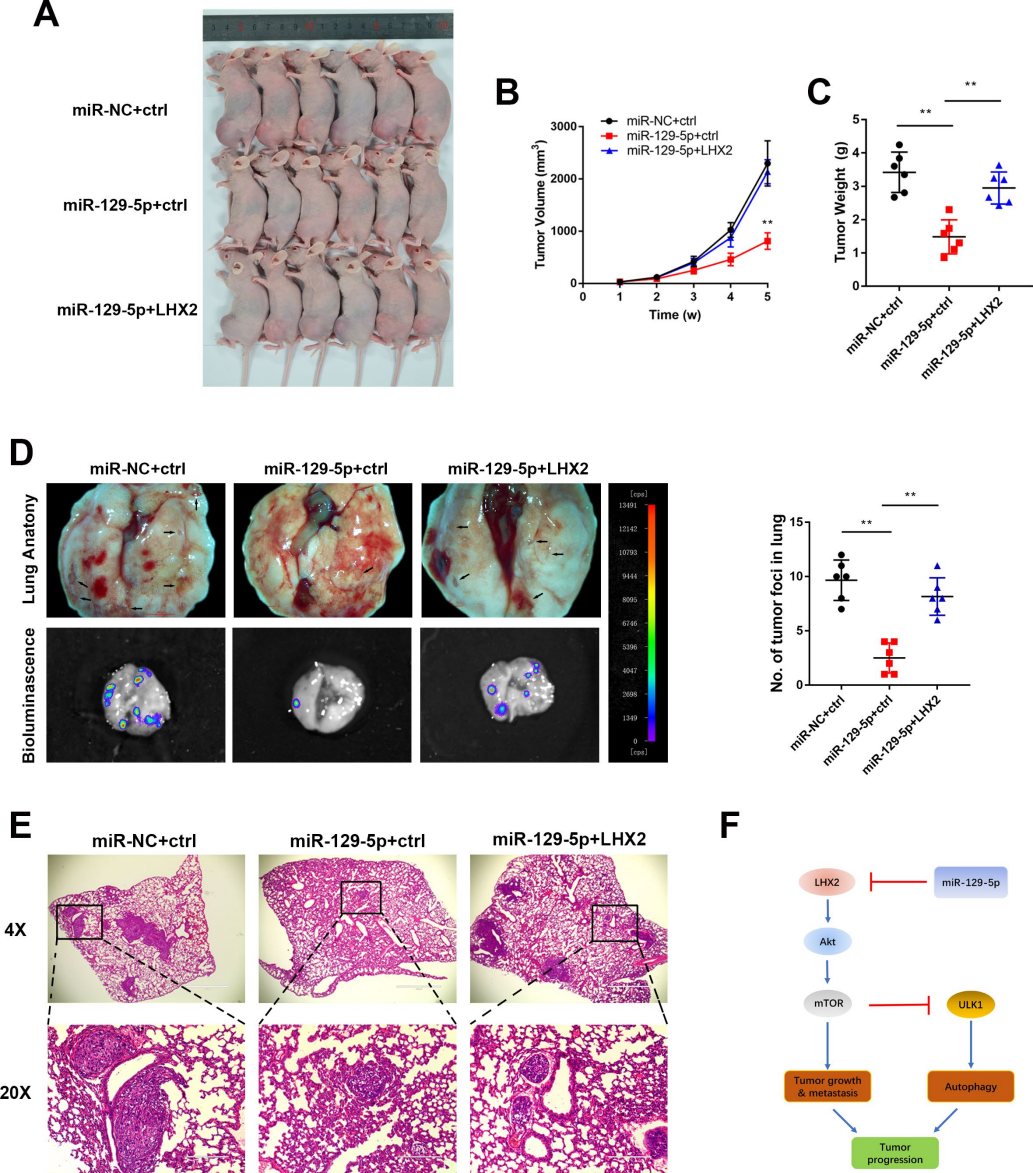
**Restoration of LHX2 reverses miR-129-5p overexpression-mediated malignant phenotypes in vivo.** (**A**) OS-143B cells stably expressed luciferase and the indicated gene were inoculated into nude mice (n=6), which were sacrificed 5 weeks later (**B**) Tumor sizes were measured weekly and calculated using the following formula: V= (Length×Width^2^/2). (**C**) Orthotopic tumors were dissected and weighted. **P<0.01. (**D**) Representative bioluminescence and lung anatomy images of metastatic OS cells. Black arrows indicate possible metastatic lesions. Metastatic foci in lung were determined by luminescence signals and counted. **P<0.01. (**E**) Representative H&E stained lung sections. (**F**) Working model of the miR-129-5p/LHX2/mTOR axis in OS.

To identify the phenotypes of LHX2 and miR-129-5p overexpression, we performed rescue experiments on tumor growth and metastasis in orthotopic xenograft models. Consistent with the findings *in vitro*, miR-129-5p overexpression remarkably inhibited tumor growth in orthotopic xenografts. However, mice inoculated with miR-129-5p that overexpressed LHX2 showed increased tumor sizes and weights (Figure 7A–7C). Bioluminescent imaging and lung anatomic analysis indicated that miR-129-5p-overexpressing groups had lower metastatic foci in the lung compared to the control group. These effects could be reversed in miR-129-5p plus LHX2 overexpression groups (Figure 7D and 7E).

## DISCUSSION

LHX2 plays an essential role in embryo development, cell fate decisions, proliferation and cell differentiation [32]. Emerging evidence demonstrates that LHX2 was up-regulated in many tumors and plays a crucial role in tumorigenesis [8–10]. Previous studies reported that LHX2 participates in the progression of breast cancer and non-small lung cancer [9, 33]. Herein, we reveal that LHX2 is up-regulated in OS and that LHX2 silencing restrains the growth and metastasis of OS *in vitro* and *in vivo*. Moreover, we found that the inhibition of LHX2 enhanced autophagy in OS cells.

Accumulating evidence has demonstrated that autophagy is required for cancer progression. On the one hand, the degradation of damaged organelles or proteins provides the energy to support cell metabolism and promote cell proliferation and metastasis [13]. On the other hand, autophagic cell death induced by chemotherapy leads to growth inhibition and reduced autophagy, which may contribute to increased multidrug resistance (MDR) [18, 34]. Herein, we found that LHX2 negatively regulates autophagy, which may contribute to tumor progression.

Multiple signaling pathways have been implicated in the proliferation and metastasis of OS, including MAPK, PI3K/Akt [35], Wnt/β-catenin [36], RhoA-ROCK-LIMK2 [37]. Kuzmanov et al. reported the increased expression of LHX2 in TGF-β-induced EMT processes in breast cancer [9], whilst others demonstrated that LHX2 activates Wnt/β-catenin signaling to promote EMT in pancreatic ductal carcinoma nasopharyngeal carcinoma [8]. Emerging evidence demonstrates that mTOR signaling is a crucial regulator of cancer progression. The abnormal activation of mTOR promotes proliferation and inhibits the apoptosis of tumor cells via activating Akt [38–40]. In this study, we found LHX2 silencing inactivated Akt and mTOR, revealing the molecular basis for how LHX2 promotes cell proliferation and metastasis in OS. Besides, mTOR is a well-known regulator of autophagy induction [21, 41], the initiation of which is associated with the activation of ULK1 complexes [42, 43]. Previous studies have shown that increased mTOR activity prevents ULK1 activation under nutrient sufficiency [23, 44]. The ULK1/FIP200 complex has been shown to promote the activation of Beclin-1 and enhance autophagy [45]. Herein, we found that the inhibition of LHX2 decreased mTOR activity and increased the expression of ULK1 and Beclin-1, suggesting that the inhibition of autophagy mediated by LHX2 occurs through the mTOR pathway.

MiRNAs regulate gene expression by targeting mRNAs [24, 46]. To explore the mechanisms of increased LHX2 expression in OS, we performed bioinformatic predictions, luciferase reporter assays, western blot analysis and q-PCRs to investigate the possible regulatory factors of LHX2. The results demonstrated that miR-129-5p negatively regulates LHX2. Emerging evidence demonstrates that miR-129-5p participates in cancer progression [47]. Our previous study demonstrated that miR-129-5p inhibits malignant phenotypes *in vitro* [30]. Herein, we confirmed that miR-129-5p suppresses OS growth and metastasis by at least in-part, targeting LHX2.

In conclusion, this study revealed that LHX2 acts as an oncogene in OS to promote malignant phenotypes via the activation of mTOR signaling and autophagy. Moreover, we found that LHX2 is a novel target of miR-129-5p and that the miR-129-5p/LHX2/mTOR axis represents a potential candidate for OS management (Figure 7F).

## MATERIALS AND METHODS

### Tissues specimens and patients

A total of 71 OS tissues were obtained from the First Affiliated Hospital of Nanchang University, China. All the patients received no preoperative chemotherapy or radiotherapy prior to biopsy. LHX2 expression was evaluated through IHC analysis. The clinical parameters are shown in Table 1. Information from 11 follow-ups was absent. The study was approved by the ethics committee of the First Affiliated Hospital of Nanchang University.

### Cell culture and reagents

The osteosarcoma cell lines 143B and MG63 were cultured in DMEM (Gibco, CA, USA). Saos-2 and hFOB 1.19 cells were cultured in McCOY’s 5A and DMEM/F12 respectively. All cells were supplemented with 10% FBS (Gibco). Anti-LHX2 (ab184337) antibodies were purchased from Abcam (Cambridge, MA, USA), anti-GAPDH (TA802519), anti-Ki67 (TA802544), anti-Beclin1 (TA502527), and anti-MAP1LC3B (TA301543) were purchased from Origene (Rockville, MD, USA). Anti-SQSTM1/P62 (88588), anti-mTOR (2972), anti-p-mTOR (2971), anti-Akt (9272), anti-p-Akt (9271), anti-ULK1 (8054), and anti-p-ULK1 (5869) were purchased from CST (Danvers, MA, USA). The autophagy inhibitor chloroquine (HY-17589) was purchased from MCE (Monmouth Junction, NJ, USA).

### R2 database analysis

The bioinformatics software R2 database (http://hgserver1.amc.nl) was performed to investigate the relationship between LHX2 and other genes. Tumor types termed mixed OS- Kuijjer - 127 -vst - ilmnhwg6v2 were selected.

### Tissues microarrays

The human osteosarcoma TMA (n=40) was purchased from Alena Biotechnology Co., Ltd (Xi'an, China). Detailed parameters of the 40 patients are listed in Supplementary Table 1.

### Lentivirus-vector construction and cell transfection

To construct vectors for LHX2 upregulation, human LHX2 (NM_004789.3) with mutant binding site of miR-129-5p was subcloned into the FV050 (CMV-MCS-3Flag-SV40-mCherry-IRES-Puromycin) vector. For shLHX2, three short hairpin RNAs were designed to silence LHX2 expression. The sequences included: shLHX2-1:5′-GCTTCGGACCATGAAGTCT TA-3′; shLHX2-2:5′-GCAACCTCTTACGGCAGGAAA- 3′; shLHX2-3:5′-CAACTGTGACGTCCGTCTTAA-3′. The results of qRT-PCR and western blot analysis indicated that shLHX2-3 showed the highest knockdown efficiency. 143B and MG63 cells were incubated with 1 × 10^6^ lentivirus-transducing units for 12 h (MOI=100). After 72 h, 0.6 and 0.8 μg/ml of puromycin were added for cell selection.

### qRT-PCR

Total RNA was extracted and reverse-transcribed into cDNA. QRT-PCR experiments for mRNA detection were performed with a ChamQTM Universal SYBR® qPCR Master Mix (Vazyme, Nanjing, China) according to the manufacturer’s instructions. GAPDH was used as a control. The miRNA Universal SYBR qPCR Master Mix (Vazyme) was used for miRNA detection. U6 was used as a control. Relative gene expression was calculated using the Comparative Ct method. Detailed information of the primer sequences is shown in Table 2.

**Table 2:** Primer sequences.

**Primer sequences for miRNA detection (by stem-loop)**		
**Name**	**Sequence**	
miR-129-5p-RT	GTCGTATCCAGTGCGTGTCGTGGAGTCGGCAATTGCACTGGATACGACGCAAGCCC	
U6-RT	GTCGTATCCAGTGCAGGGTCCGAGGTATTCGCACTGGATACGACAAAAAT	
miR-129-5p- F	GGCTTTTTGCGGTCTGG	
miR-129-5p- R	CAGTGCGTGTCGTGGAGT	
U6-F	CTCGCTTCGGCAGCACA	
U6-R	AACGCTTCACGAATTTGCGT	
**Primer sequences for mRNA detection**		
**Name**	**Forward (5′→3′)**	**Reverse (5′→3′)**
LHX2	TTCCAGAACGCCCGAGCCAA	GGGGCTAGTCAAGTCTGTC
GAPDH	CCACCCATGGCAAATTCCATGGCA	TCTAGACGGCAGGTCAGGTCCACC
LC3B	TCGCCGACCGCTGTAA	AAGCCGTCCTCGTCTTTCT
BECN1	CTCCCGAGGTGAAGAGCATC	AATGGAGCTGTGAGTTCCTGG
ATG3	AAGTGGCTGAGTACCTGACC	GATCTCCAGCTGCCACAAAC
ATG7	GAACAAGCAGCAAATGA	GACAGAGGGCAGGATAG
ATG12	TTGCTAAAGGCTGTGGGAGA	ACTGTTCTGAGGCCACAAGT
LAMP1	CTGCTGCCTTCTCAGTGAAC	TCTGATGGCAGGTCAAAGGT

### Western blot analysis

For western blot analysis, cells were lysed with RIPA buffer containing 1% protease cocktail. Proteins were electrophoresed on 10%-12% SDS-PAGE gels and transferred onto PVDF membranes (Millipore, Darmstadt, Germany). Membranes were blocked in 5% skimmed milk (BD Biosciences, CA, USA), followed by incubation with primary antibodies for 8 h. Membranes were washed 3 times in 1× TBST for 15 min, and anti-mouse (Abcam) or anti-rabbit (Abcam) secondary antibodies were added. Immunocomplexes were visualized after incubation with ECL reagents (Amersham Biosciences, Piscataway, NJ, USA) using a digital gel image analysis system (TANON, Japan).

### Dual-luciferase report assays

Reporter constructs containing putative or mutant 3′-UTRs of LHX2 were cotransfected with miR-129-5p into 143B and MG63 cells with Lipofectamine 3000 (Thermo Fisher Scientific, MA, USA). After 72 h, cells were collected to examine luciferase activity as previously described [48].

### Cell proliferation and colony formation assays

The OS cell lines 143B or MG63 were seeded into 96-well plates for 24 h, 48 h, 72 h, and 96 h. CCK8 reagents (Dojindo Laboratories, Kumamoto, Japan) were incubated for 2 h and cell viability was measured 450 nm. For colony formation assays, ~1000 cells were seeded into 6-well plates for 7 days and then fixed with PFA (4%) and stained with crystal violet (2%). Colonies were defined as clusters of more than 50 cells and counted.

### Transwell assays

Briefly, 3×10^5^ cells were resuspended in 150 μl of serum-free medium and seeded into 8 μm chambers (Millipore) pre-coated with or without Matrigel (1:8 dilution; BD). Chambers were incubated in 500 μl of complete medium for 24 h. Cotton swabs were used to remove the remaining Matrigel and cells in the upper chamber were stained. Chambers were soaked in 4% PFA and crystal violet-stained. Cells in 6 microscopic fields were counted and imaged (10× magnification).

### Wound healing assays

Briefly, 6×10^6^ 143B and MG63 cells were plated into six-well plates and grown to 90% confluency. Scratch assays were performed with a 10 μl plastic pipette tip. Images of the wound were taken at 0 h and 24 h. Cell migration distances were calculated using ImageJ and compared to T=0.

### GFP-RFP-LC3 fusion assays

143B cells stably expressing the GFP-RFP-LC3B reporter were treated with vehicle (DMSO) for the indicated times. Laser scanning confocal microscope (ZEISS/LSM 800, Germany) was used to image changes in fluorescence.

### Spontaneous metastasis xenografts

Female Balb/c nude mice (4~6 weeks old) were purchased from the Nanjing BioMedical Research Institute of Nanjing University (NBRI) and housed in the SPF Transgenic Animal Facility of Nanchang University. All animal procedures were approved by the Institutional Animal Care and Use Committee of Nanchang University. Orthotopic spontaneous metastasis/orthotopic OS animal models were established using 143B cells stably expressed firefly luciferase and transfected with the indicated lentiviruses to alter relative gene expression. Cells were cultured for 10 generations. Approximately 1×10^7^ cells (resuspended in 100ul DMEM) Luc-143B cells were subcutaneously injected into anesthetized nude mice. Two weeks later, subcutaneously grown tumors were collected and sectioned into 2-3 mm fragments. A single fragment was transplanted into the left tibia of 6-8 weeks-old anesthetized nude mice and covered with bone wax (Braun, Germany). Tumor sizes were measured weekly using a caliper. Mice were sacrificed 5 weeks after transplantation and orthotopic tumors were dissected and fixed in 10% formalin after weighing. Lung tissues were dissected and the foci of pulmonary metastasis were detected by bioluminescent imaging. Lung tissues were then fixed in formalin for further detection. For bioluminescent imaging, D-luciferin (150mg/kg) (yuanyebio, shanghai, China) was injected into the anesthetized mice prior to imaging. Bioluminescent images were acquired using the Night OWL LB 983 Imaging System (Berthold, Germany).

### Histology and IHC

For histopathological assays, paraffin-embedded tissues were fixed in 10% formalin, and sectioned to a thickness of ~3 μm. Sections were stained with 4% hematoxylin for 15s and eosin for 1s (H&E). Sections were imaged on an BX63 Olympus microscope (Olympus). For immunohistochemistry (IHC) assays, sections were treated as previous described [49] and probed with anti-LC3B and anti-Ki67 antibodies (1:100 dilution) at 4 °C overnight. Sections were labeled with the appropriate secondary antibodies for 20 min using Histostain Plus kits (Invitrogen, CA, USA). IHC expression of LC3 and Ki67 were determined by two pathologists blinded to the specimens.

### Statistical analysis

Data from the GEO database were analyzed using the non-parametric Wilcoxon rank-sum test. All measurements are shown as the mean ± standard deviation. A student’s t-test was used for two-sample analysis. A one-way ANOVA was used for multiple-sample analysis. All analyses were performed using SPSS statistical software version 23.0 (SPSS, Inc. Chicago, IL).

## Supplementary Material

Supplementary Figure 1

Supplementary Table 1
