# Supplementary material for: LHX2 promotes malignancy and inhibits autophagy via mTOR in osteosarcoma and is negatively regulated by miR-129-5p
Source: Aging (Albany NY). 2019 Nov 13;11(21):9794–810. doi: 10.18632/aging.102427 (PMC6874432; doi:10.18632/aging.102427)
Supplement: Supplementary Figure 1 [file aging-11-102427-s002.pdf]

SUPPLEMENTARY FIGURE

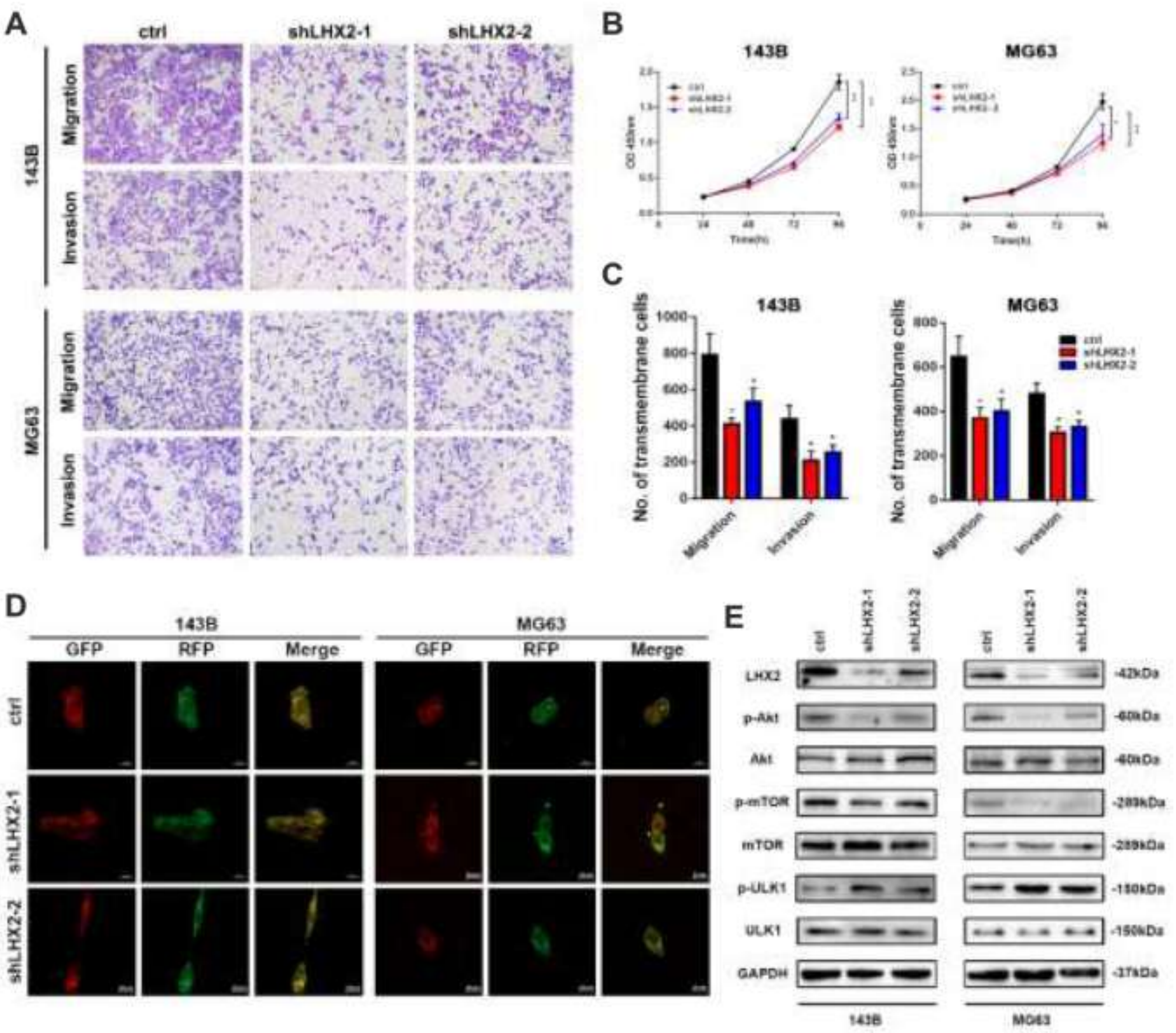

Supplementary Figure 1. LHX2 silence inhibits the OS malignancy and enhances autophagy via mTOR. (**A**–**C**) Transwell migration, invasion, and CCK8 assays were carried out to to detect the malignancy in 143B and MG63 cells transfected with shLHX2-1, shLHX2-2 and control group. Significant differences were determined using unpaired Student's t test. *P <0.05, **P<0.01. Scale bar: 100 µm. (**D**) 143B and MG63 cells transfected with shLHX2-1, shLHX2-2 and control group were scoped by confocal. Red puncta: autolysosomes; Yellow puncta: autophagosomes. Scale bar: 20 µm. (**E**) Western blots of LHX2, p-Akt (Ser473), Akt, p-mTOR (Ser2448), mTOR, p-ULK1 (Ser757), ULK1 in 143B and MG63 cells transfected with shLHX2-1, shLHX2-2 and control group. GAPDH was used as endogenous control.
